# Inflammatory Cell Recruitment in *Candida glabrata* Biofilm Cell-Infected Mice Receiving Antifungal Chemotherapy

**DOI:** 10.3390/jcm8020142

**Published:** 2019-01-26

**Authors:** Célia F. Rodrigues, Alexandra Correia, Manuel Vilanova, Mariana Henriques

**Affiliations:** 1 LIBRO – ‘Laboratório de Investigação em Biofilmes Rosário Oliveira’, Centre of Biological Engineering, University of Minho, 4710-057 Braga, Portugal; mcrh@deb.uminho.pt; 2 Laboratory for Process Engineering Environment Biotechnology and Energy-Department of Chemical Engineering, Faculty of Engineering, University of Porto, 4200-465 Porto, Portugal; 3 Instituto de Investigação e Inovação em Saúde, Universidade do Porto, 4200-135 Porto, Portugal; alexandra.correia@ibmc.up.pt (A.C.); vilanova@icbas.up.pt (M.V.); 4 Instituto de Biologia Molecular e Celular, Universidade de Porto, 4200-135 Porto, Portugal; 5 Departamento de Imuno-Fisiologia e Farmacologia, Instituto de Ciências Biomédicas de Abel Salazar, Universidade do Porto, 4050-313 Porto, Portugal

**Keywords:** *Candida glabrata*, candidemia, echinocandins, resistance, biofilms, infection, micafungin, caspofungin, *in vivo*

## Abstract

(1) Background: Due to a high rate of antifungal resistance, *Candida glabrata* is one of the most prevalent *Candida* spp. linked to systemic candidiasis, which is particularly critical in catheterized patients. The goal of this work was to simulate a systemic infection exclusively derived from *C. glabrata* biofilm cells and to evaluate the effectiveness of the treatment of two echinocandins—caspofungin (Csf) and micafungin (Mcf). (2) Methods: CD1 mice were infected with 48 h-biofilm cells of *C. glabrata* and then treated with Csf or Mcf. After 72 h, the efficacy of each drug was evaluated to assess the organ fungal burden through colony forming units (CFU) counting. The immune cell recruitment into target organs was evaluated by flow cytometry or histopathology analysis. (3) Results: Fungal burden was found to be higher in the liver than in the kidneys. However, none of the drugs was effective in completely eradicating *C. glabrata* biofilm cells. At the evaluated time point, flow cytometry analysis showed a predominant mononuclear response in the spleen, which was also evident in the liver and kidneys of the infected mice, as observed by histopathology analysis. (4) Conclusions: Echinocandins do not have a significant impact on liver and kidney fungal burden, or recruited inflammatory infiltrate, when mice are intravenously (i.v.) infected with *C. glabrata* biofilm-grown cells.

## 1. Introduction

*Candida glabrata* is one of the most common causes of systemic fungal infection (candidemia), surpassed only by *Candida albicans* [1,2,3]. It is the second most common isolated yeast in the United States of America and the third in Europe, after *Candida parapsilosis*, accounting for 20% of candidemia [2,4]. As a commensal yeast, *C. glabrata* colonizes and adapts to many different niches in the human body and can be isolated from the mucosae of healthy individuals [2,5]. Yet, as an opportunistic pathogen, this fungus can also be the point of origin for mucosal infections and severe candidemia. Its biofilm-forming ability and the ability to rapidly acquire resistance to antifungals (especially to azoles) [2,5,6], which in many cases can be further increased by genetic and genomic mutations (e.g., polymorphisms, the formation of new chromosomes, karyotype variations) [7,8,9], may contribute to increased virulence. 

Risk factors for the development of invasive *C. glabrata* infections in human patients comprise immunosuppression (e.g., cancer chemotherapy, human immunodeficiency virus (HIV) infection, diabetes mellitus, neutropenia), mucosal colonization by *Candida* spp., the use of indwelling medical devices (e.g., vascular catheters), and gastrointestinal surgery [10,11,12]. 

During infection, *C. glabrata* virtually colonizes all sites and organs, which reveals a high capacity to adapt to the many different niches inside the human host [1]. Oral and systemic *C. glabrata* infections have high associated morbidity and mortality [13,14,15] and the rise in incidence infections caused by this yeast is to some extent attributable to its ability to tolerate or resist many antifungals commonly used in clinical practice [2,16,17]. The occurrence of oral candidiasis related to *C. glabrata* is increasing [15,18]. Although *C. glabrata* colonization does not always lead to infection, it is a foreword to infection when the risk of systemic infection is elevated, or the host immunity is compromised. *C. glabrata* infections are a major challenge [15,19,20]. The good biofilm-forming ability and raised enzymatic activity of *C. glabrata* are two of the most important features favoring oral and systemic candidiasis. In fact, biofilms can be formed on both biotic (e.g., gastrointestinal or mouth mucosae) and abiotic surfaces (e.g., indwelling medical devices) [21,22] and biofilm cells are recognized to be more resistant to antifungal treatment than planktonic cells, as well as responsible for more severe infections [2,23,24,25]. Systemic candidiases are the most prevalent invasive mycoses worldwide with mortality rates close to 40% and *C. glabrata* is frequently recognized as a causative agent [26]. In nearly all these cases, the infections are related to the use of a medical device and biofilm formation on its surface [20]. The contamination of medical devices (mostly catheters) or infusion fluids can occur from the skin of the patient, the hands of health professionals [27], or by migration into medical devices from a previous lesion. Less commonly, *Candida* spp. that commensally colonize the gastrointestinal tract switch to having a pathogenic behavior, being able to infiltrate the intestinal mucosa, disseminate through the bloodstream, and colonize medical devices endogenously (this is more common in cancer patients, since chemotherapy harms the mucosa) [28]. Depending on the clinical situation, the removal of medical devices can be recommended in patients with disseminated *Candida* spp. infection to enable pathogen eradication and to improve the prognosis [29,30]. In contrast, experimental intravenous infection of laboratory animals with *C. glabrata* does not usually cause mortality, since it appears that this species has successfully developed immune evasion strategies enabling it to survive, disseminate, and persist within mammalian hosts [1,31].

Because of the high probability of innate resistance to azoles, echinocandins are recommended as first-line therapy against *C. glabrata* candidemia [32]. Nonetheless, and worryingly, *C. glabrata* is the first *Candida* spp. for which resistance to echinocandins has been identified and described [33,34]. Recently, case reports of echinocandin-resistant *C. glabrata* subsequent to different echinocandin therapies are becoming more common [35,36,37,38,39,40,41]. Indeed, one third of those isolates may be multidrug resistant [42] and have specific mutations in one of two “hot spot” regions of the *FKS1* or *FKS2* (1,3-β-glucan synthase) genes, which encode a subunit of the β-1,3-d glucan synthase protein, a target of echinocandins [35,43,44,45].

Therefore, in this work, a simulation of a hematogenously disseminated *C. glabrata* infection derived exclusively from biofilm cells (as occurs in catheter infections) was performed. CD1 mice were infected with 48 h-biofilm cells of the wild type *C. glabrata* strain ATCC2001, and then treated with the echinocandins caspofungin (Csf) and micafungin (Mcf) in order to evaluate organ fungal burdens after 72 h, the efficacy of each drug after two administrations, and the associated inflammatory response.

## 2. Experimental Section

### 2.1. Ethics Statement 

This study was performed in strict accordance with the recommendations of the European Convention for the Protection of Vertebrate Animals used for Experimental and Other Scientific Purposes (ETS 123), the 86/609/EEC directive, and Portuguese rules (DL 129/92). All experimental protocols were approved by the competent national authority (Direcção-Geral de Veterinária), document 0420/000/000/2010. Female CD1 mice, 8–12 weeks old, were purchased from Charles River (Barcelona, Spain) and kept under specific pathogen-free conditions at the Animal Facility of the Instituto de Ciências Biomédicas Abel Salazar, Porto, Portugal. Mice were maintained in individually ventilated cages (five animals per cage) with corncob bedding, and under controlled conditions of temperature (21 ± 1 °C), relative humidity (between 45 and 65%), and light (12 h light/dark cycle). Mice had ad libitum access to food and water. Hiding and nesting materials were provided for enrichment. All procedures such as cage changing, water and food supply, as well as intravenous and intraperitoneal injections were always performed during the day cycle (between 7 a.m. and 7 p.m.).

### 2.2. Organisms and Growth Conditions

One strain of the American Type Culture Collection (ATCC), *C. glabrata* ATCC2001, was subcultured on Sabouraud dextrose agar (SDA) (Merck, Darmstadt, Germany) for 24 h at 37 °C. Cells were then inoculated in Sabouraud dextrose broth (SDB) (Merck, Darmstadt, Germany) and incubated for 18 h at 37 °C under agitation at 120 rpm. Biofilms were formed in 24-well polystyrene microtiter plates (Orange Scientific, Braine-l’Alleud, Belgium) [46]. For this, 1000 µL of the yeast cell suspension (1 × 10^5^ cells/mL) was added to each well and incubated for 24 h. After 24 h, 500 µL of RPMI 1640 was removed and an equal volume of fresh medium was carefully added. Biofilms allowed to grow, under the same temperature and agitation conditions, for an additional 24 h. After this time (total 48 h), all media were removed and the biofilms carefully washed to remove non-adhered cells. Biofilms were scraped from the 24-well plates, resuspended in ultra-pure water, sonicated (Ultrasonic Processor, Cole-Parmer, IL, USA) for 30 s at 30 W, and then suspension vortexed for 2 min. The suspension was centrifuged at 5000 *g* for 5 min at 4 °C, as previously optimized [46,47]. The pellets of the biofilm cells were then suspended in RPMI 1640 and the cellular density was adjusted to 5 × 10^8^ cells/mL using a Neubauer counting chamber. 

### 2.3. Antifungal Drugs

Csf and Mcf were kindly provided by MSD^®^ and Astellas^®^, respectively. Aliquots of 5000 mg/L were prepared using dimethyl-sulfoxide (DMSO). The final concentrations used were prepared with pyrogen-free phosphate buffer saline (PBS) for both drugs.

### 2.4. Murine Model of Hematogenously Disseminated Infection

*Candida glabrata* inoculum was prepared following previously described procedures [47,48]. The number of cultivable cells was assessed by colony forming units (CFU) counting and were injected intravenously in the lateral tail vein, with the support of a restrainer. Sample size was determined based on the results of preliminary experiments. On day 0, adult CD1 mice, randomly allocated to each experimental group, received 200 μL of *C. glabrata* biofilm cell suspensions containing 5 × 10^8^ CFU i.v. via the tail vein. Control mice were injected intravenously with 200 μL of pyrogen-free PBS. Treatment with the echinocandins started 24 h post-inoculation and was administered intraperitoneally (i.p.) with a volume of 0.5 mL at 24 and 48 h post-inoculation. Doses were as follows: caspofungin 6 mg/kg and micafungin 12 mg/kg. This experimental scheme (days and dosages) were chosen on the basis of previous pharmacodynamic studies of echinocandins against *C. glabrata* and a need to reach drug exposures in mice that were comparable to those in humans receiving currently licensed echinocandin regimens [32,49,50]. Liver and kidneys were aseptically removed, weighed, homogenized, and quantitatively cultured on Sabouraud dextrose agar (Difco) at 37 °C. Values are expressed as log CFU per gram of liver. Two independent experiments were performed, with at least five animals per infected group.

### 2.5. Flow Cytometry 

For flow cytometry analysis, spleens from infected mice and controls were aseptically removed 72 h post-infection, homogenized in Hanks’ Balanced Salt Solution (Sigma Aldrich, Roswell-Park, St. Louis, MO, USA) and, when necessary, red blood cells were lysed. The following monoclonal antibodies (mAb) were used (at previously determined optimal dilutions) for surface antigen staining after pre-incubation with anti-mouse CD16/CD32 for FcγR blocking. For dead cell exclusion, all samples except single-stained controls were first incubated with allophycocyanin (APC) eFluor 780 Fixable Viability Dye (eBioscience, San Diego, CA, USA) diluted 1:1000 in PBS for 30 min at 4 °C. For surface staining, cells were incubated with the following monoclonal antibodies: anti-mouse GR1 Fluorescein isothiocyanate (FITC)-conjugate, anti-mouse CD80 Phycoerythrin (PE)-conjugate, anti-mouse F4/80 Peridinin-chlorophyll protein Cyanin 5.5 (PerCp Cy5.5)-conjugate, anti-mouse CD86 PE-cychrome 7 (PE-Cy7)-conjugate, anti-mouse CD11c BV421-conjugate (all from BD Biosciences, San Jose, CA, USA), anti-mouse CD11b BV510-conjugate, and anti-mouse major histocompatibility complex (MHC) class II APC conjugate (eBiosciences, San Diego, CA, USA). Data acquisition was performed in a FACSCanto^TM^ II system (BD Biosciences, San Jose, CA, USA) using the FACSDIVA^TM^ software (BD) and compensated and analyzed in FLOWJO version 9.7.5. (Tree Star Inc., Ashland, OR, USA). A biexponential transformation was applied to improve data visualization; 10^6^ cells were stained per sample. 

### 2.6. Histopathologic Examination and Immunohistochemistry

Livers were fixed in buffered formalin and embedded in paraffin for hematoxylin-eosin (HE) and periodic acid–Schiff (PAS) histopathologic analysis, as previously described [51,52]. 

### 2.7. Statistical Analysis

Statistical analysis was carried out with Prism^TM^ 7 (GraphPad^TM^, San Diego, CA, USA). The normality of the data obtained was evaluated using the Kolmogorov–Smirnov test. Accordingly, Kruskal–Wallis and Sidak’s multiple comparison tests were applied and data were depicted as means of all independent experiments. Differences among groups were considered significant when *P* < 0.05. 

## 3. Results and Discussion

Candidemia has been increasing in the last decades, especially among individuals under chemotherapy programs, as well as in those who are HIV-positive, hospitalized, or catheterized [2,53]. *C. albicans* is still the most frequent isolated yeast, but *C. glabrata* has become one of the most threatening non-*Candida albicans Candida* (NCAC) spp., mostly due to its high antifungal resistance [2,54]. Though human clinical data demonstrate that immunosuppression is a risk factor for *C. glabrata* infections, it is not an absolute prerequisite for *C. glabrata* candidiasis [55]. Hence, increasing the data on the host immune response to *C. glabrata* and revising the efficacy of chemotherapeutic approaches to treat infections caused by this fungus are of major value. The murine model is a suitable one to address both issues, alone or combined [56].

### 3.1. Fungal Burden Progression Differs Substantially between Liver and Kidneys

The fungal burden of CD1 mice infected intravenously with *C. glabrata* biofilm cell suspensions and subsequently treated with echinocandins was assessed in the liver and kidneys 72 h post-infection. No differences were observed among the different infected groups.

**Figure 1:**
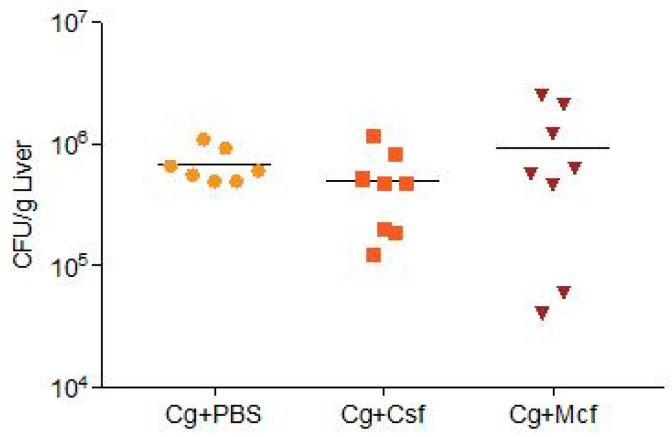
Liver fungal burden of CD1 mice 72 h after intravenously challenged with 1 × 10^8^ biofilm cells plus two cycles of treatment with PBS, caspofungin (Csf), or micafungin (Mcf). Data are representative of two independent experiments. Each symbol represents an individual mouse, and horizontal bars are means of colony forming unit (CFU) numbers for each group. The obtained results are displayed as CFU/liver. Controls (naïve; PBS + Csf; PBS + Mcf), *n* = 2; Cg + Csf, *n* = 8; Cg + Mcf, *n* = 8. No statistical differences were observed among infected groups (evaluated by Kruskal–Wallis (Overall ANOVA *P* < 0.05) and post hoc Sidak’s multiple comparison tests). Cg—*Candida glabrata* ATCC2001.

In contrast to *C. albicans*, which can heavily infect the kidneys [57], a tropism of *C. glabrata* to the liver was clearly noticed. High CFU counts were detected on this organ (Figure 1), in contrast to the low or non-detected CFU counts in the kidneys (≤3 × 10^4^ CFU/g kidney). The low colonization of this organ, as compared to the liver or brain in immunocompetent mice systemically infected with *C. glabrata*, was also reported by other authors [1,31,58,59,60]. Nevertheless, Kaur et al. [59], Srikantha et al. [60], and Brieland et al. [58] stated that *C. glabrata* could be recovered after several days in the kidneys, liver, spleen, hearts, lungs, brains, and lungs. Moreover, Atkinson et al. [61] described that fungal burdens were 10^4^ to 10^8^ in immunocompromised mice in the spleen and kidneys. Nonetheless, it is important to stress that the differences in mouse strains and immunocompetence status, *C. glabrata* strains, animal age and gender, or even the concentration of the inoculum used do not allow a direct comparison of published data [31]. In addition, past *in vitro* reports have shown that susceptible *C. glabrata* strains can become resistant in less than four days of continuous culture with low doses of drugs, such as fluconazole [1,16] and echinocandins [62,63,64,65]. Thus, it is plausible that a fast increase of resistance could have been observed in vivo. Moreover, the inoculum exclusively contained biofilm cells, known to be more resistant than their planktonic counterparts [66,67,68,69,70,71,72].

### 3.2. Host Immune Response to Hematogenously Disseminated Candidiasis 

In contrast to the considerable work that has been described on the host immune response to *C. albicans*, the immune mechanisms elicited in the course of *C. glabrata* infections are far less explored.

**Figure 2:**
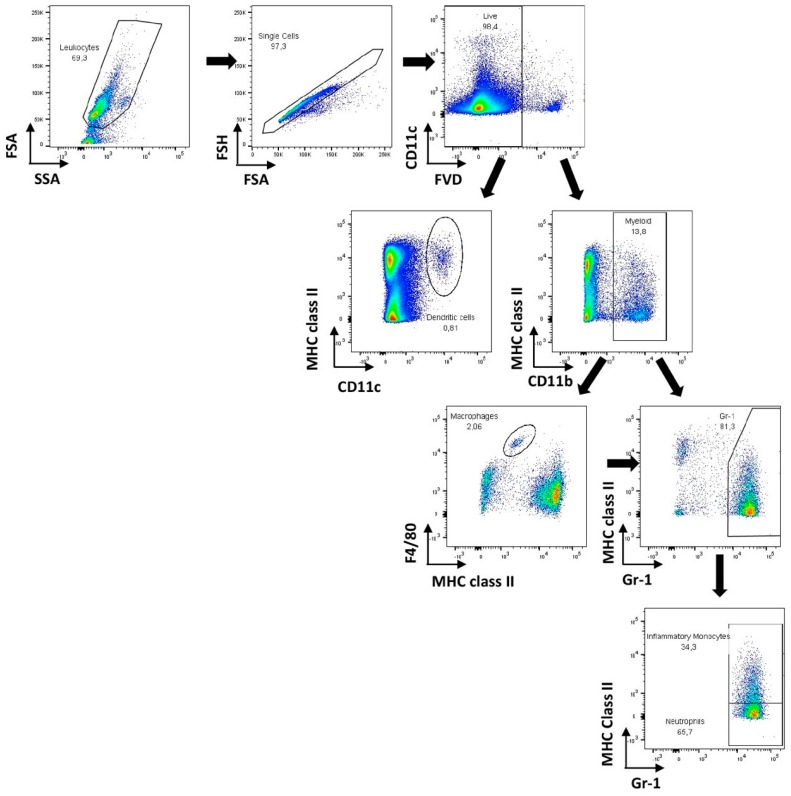
Gating strategy applied for the flow cytometry data analysis. Following leukocyte selection based on Forward Scatter Area (FSA) and Side Scatter Area (SSA), doublets were excluded based on FSA and Forward Scatter Height (FSH) parameters, and dead cells were further excluded by fixable viability dye (FVD) incorporation. Dendritic cells were gated as CD11c^high^ MHC class II^+^ cells. Myeloid cells were defined as CD11b^+^ cells that were further divided into macrophages (CD11b^+^ F4/80^high^ MHC class II^low^) and Gr-1^+^ cells. Within the latter, neutrophils were defined as CD11b^+^ Gr-1^+^MHC class II^−^ and inflammatory monocytes were gated as CD11b^+^ Gr-1^+^MHC class II^+^ cells.

**Figure 3:**
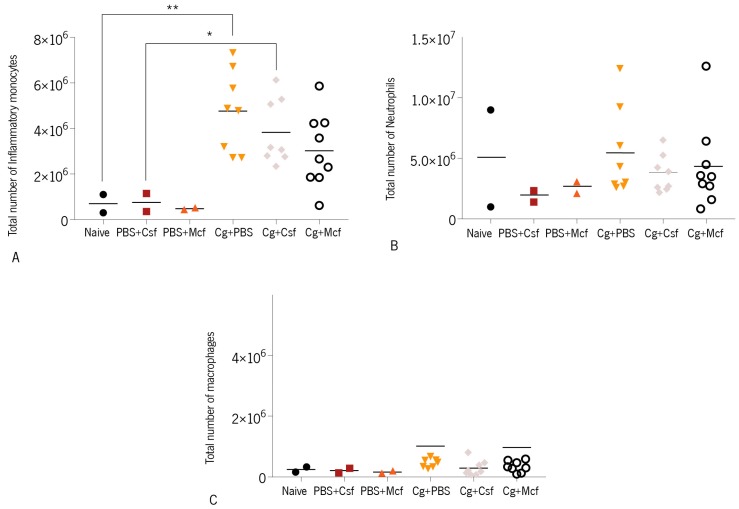
CD1 mice were challenged intravenously with 1 × 10^8^ biofilm cells and then treated with PBS, caspofungin (Csf), or micafungin (Mcf). The obtained results are displayed as the total number of cells of indicated populations: (**A**) inflammatory monocytes, (**B**) neutrophils, and (**C**) macrophages. The numbers of animals used were as follows: controls (naïve; PBS + Csf; PBS + Mcf), *n* = 2; Cg + Csf, *n* = 8; Cg + Mcf, *n* = 8. Statistical differences were evaluated using Kruskal–Wallis and post hoc Sidak’s multiple comparison tests (Overall ANOVA *P* < 0.05). Cg—*Candida glabrata* ATCC2001. * *P* < 0.05; ** *P* < 0.001.

Neutrophils and macrophages are in the first line of host immune defence against *Candida* spp. cells infecting the bloodstream or the endothelia [73,74,75]. Clinical observations and experimental studies have demonstrated the main role of polymorphonuclear leukocytes in mediating host protection against systemic *C. albicans* infections [76,77,78]. In mice, neutrophils have a Gr-1^high^ surface phenotype and macrophages typically express the F4/80 cell surface marker. Previous reports have shown that, in *C. albicans* infections*,* Gr-1^+^ splenocytes may have immunosuppressive function and F4/80^+^ cells may play a pro-inflammatory role [79,80]. The expression of these two surface markers was analyzed using flow cytometry in the spleen of CD1 mice 72 h after i.v. infection with 1 × 10^8^ *C. glabrata* biofilm cells. Myeloid cells (CD11b^+^) displaying the phenotypes F4/80^high^ Gr-1^neg^, F4/80^high^ Gr-1^high^, and F4/80^neg/low^ Gr-1^high^ were respectively considered macrophages, inflammatory monocytes, and neutrophils [81]. The gating strategies employed in this study are shown in Figure 2. As shown in Figure 3A, a significant increase in the numbers of inflammatory monocytes was observed in the spleen of infected mice, while those of neutrophils and macrophages remained within control values. No significant differences, however, were observed among treated groups. These results are in accordance with previous reports [31,82,83]. Unlike *C. albicans* infections, for which high neutrophil infiltration is a commonly observed feature, *C. glabrata* infections are not associated with massive neutrophil infiltration. Indeed, *C. glabrata* infection has mainly been associated with mononuclear cell infiltration and is far less inflammatory. One of the reasons given to explain this disparate outcome is that *C. albicans* hyphae cause significant host cell damage, which results in the extensive recruitment of myeloid cells and the production of pro-inflammatory cytokines [31,82,83]. 

Additionally, other reports have shown that *C. glabrata* is recognized and phagocytized by macrophages at a much higher rate than *C. albicans* [84]. After recognizing pathogens, macrophages release cytokines that help coordinate the immune response. However, when *C. glabrata* is internalized by macrophages, it interferes with the phagosome maturation process [85], surviving through autophagy and replicating inside the phagosome until the eventual bursting of the phagocyte [59,85,86]. Here, no elevated numbers of macrophages were detected in the spleen of infected mice as compared to noninfected controls (Figure 3C), which indicates that the recruitment or local proliferation of these cells does not occur in response to *C. glabrata*. 

**Figure 4:**
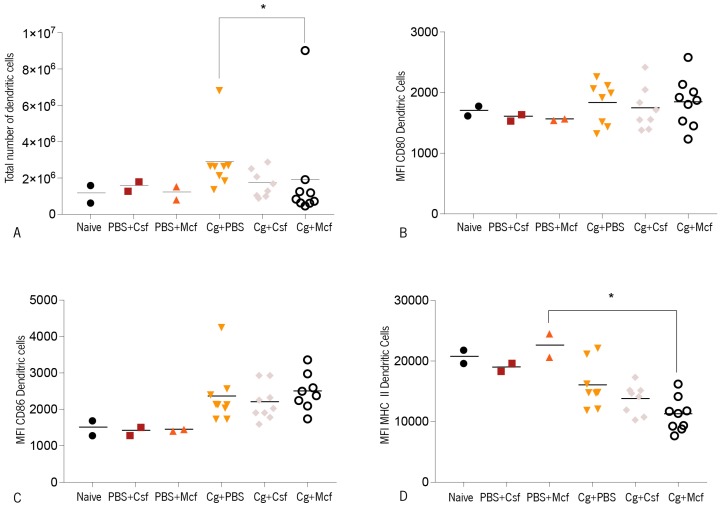
CD1 mice were challenged intravenously with 1 × 10^8^ biofilm cells and then treated with PBS, caspofungin (Csf), or micafungin (Mcf). The obtained results are displayed as (**A**) total number of dendritic cells or mean fluorescence intensities (MFI) due to antibody staining against (**B**) CD80, (**C**) CD86, and (**D**) MHC class II on the surface of dendritic cells. The numbers of animals used were: controls (naïve; PBS + Csf; PBS + Mcf), *n* = 2; Cg + Csf, *n* = 8; Cg + Mcf, *n* = 8. Statistical differences among infected groups were evaluated using Kruskal–Wallis (overall ANOVA *P* < 0.05), post hoc Sidak’s, and Dunn’s multiple comparisons tests (* *P* > 0.05). Cg—*Candida glabrata* ATCC2001. * P < 0.05.

In addition to macrophages, dendritic cells (DC) play a major role in the induction of the T cell-mediated immune response to *Candida* spp. infections [86,87] and may determine the infection outcome [88,89]. DC are able to modulate adaptive responses, depending on the *Candida* spp. morphotype encountered [73,74,90]. DC can initiate and shape the antimicrobial immunity and, since candidiasis appears frequently in immunocompromised patients, these cells may hold the key to new antifungal strategies [91]. Accordingly, the numbers of splenic conventional DC, defined as CD11c^high^ cells, and surface maturation markers were evaluated upon *C. glabrata* systemic infection (Figure 4). A slight increase in splenic DC as compared to noninfected controls was observed in the infected mice, indicating that *C. glabrata* promoted the mobilization of these cells to the spleen or promoted their local proliferation. DC surface expression of the costimulatory molecule CD86, as evaluated by the mean fluorescence intensities (MFIs) due to antibody staining (Figure 4A,B), was elevated in infected mice, showing that *C. glabrata* induced the maturation of these cells. However, the stimulatory effect was not different among the treated and nontreated groups.

In contrast, the expression of MHC class II molecules on the surface of splenic DC of mice infected with this yeast was found to be below control levels, an effect that reached statistical difference in mice treated with caspofungin. As CD86 expression in infected mice was found to be elevated, it is unlikely that this could represent a suppressive mechanism and could just be subsequent to a previous stimulatory effect. A kinetic study would be necessary to elucidate this point. 

**Figure 5:**
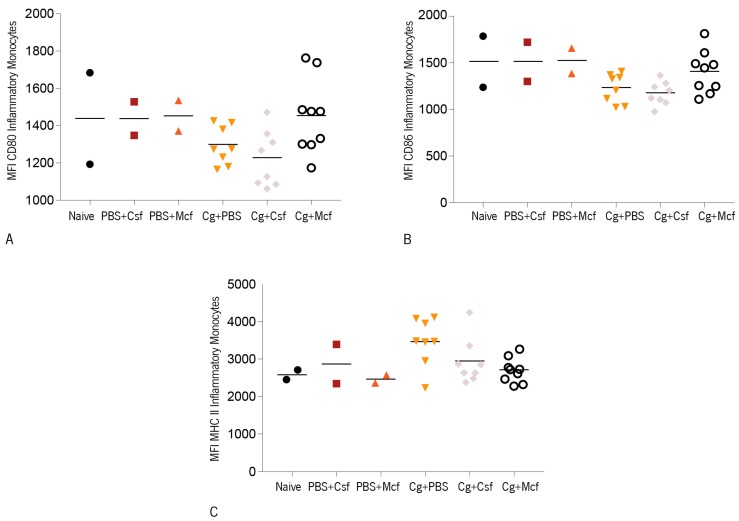
CD1 mice were challenged intravenously with 1 × 10^8^ biofilm cells and then treated with PBS, caspofungin (Csf), or micafungin (Mcf). The obtained results are displayed as mean fluorescence intensities (MFI) due to antibody staining against (**A**) CD80, (**B**) CD86, and (**C**) MHC II on inflammatory monocytes. The numbers of animals per group were: controls (naïve; PBS + Csf; PBS + Mcf), *n* = 2; Cg + Csf, *n* = 8; Cg + Mcf, *n* = 8. Statistical differences among infected groups were evaluated using Kruskal–Wallis (Overall ANOVA *P* < 0.05) and post hoc Sidak’s multiple comparison tests. Cg—*Candida glabrata* ATCC2001.

The expression of CD80, CD86, and MHC class II molecules on the surface of inflammatory monocytes was observed to be similar or slightly lower in the infected groups as compared to noninfected controls (Figure 5A–C). Likewise, and as observed on DC, no differences were observed among infected mouse groups, indicating that the treatment did not affect the expression of these activation markers on these innate immune cell populations. Finally, liver and kidney histopathologies were analyzed in infected mice, as these organs are preferred targets in i.v. *Candida* spp. infections [31,92]. As could be expected, no yeasts were found in the non-challenged control groups, and their organs presented no significant histological alterations.

Challenged mice showed inflammatory infiltrates in the liver. They were also shown, albeit less markedly, in the kidneys (nontreated and treated groups). The presence of polymorphonuclear cells was observed, but in general the infiltration remained mostly mononuclear. Yeasts were found in the liver, but not in the kidneys of treated and nontreated challenged groups. This fact corroborated the low CFU counts found in the kidneys. 

Together, these observations confirmed *C. glabrata* as a low inflammatory species and indicated that two-dose treatment with caspofungin and micafungin does not have a significant impact on liver and kidney fungal burden or recruited inflammatory infiltrate when mice are i.v. infected with *C. glabrata* biofilm-grown cells. These results confirm the biofilm in vitro outcome our group previously reported [93,94].

**Figure 6:**
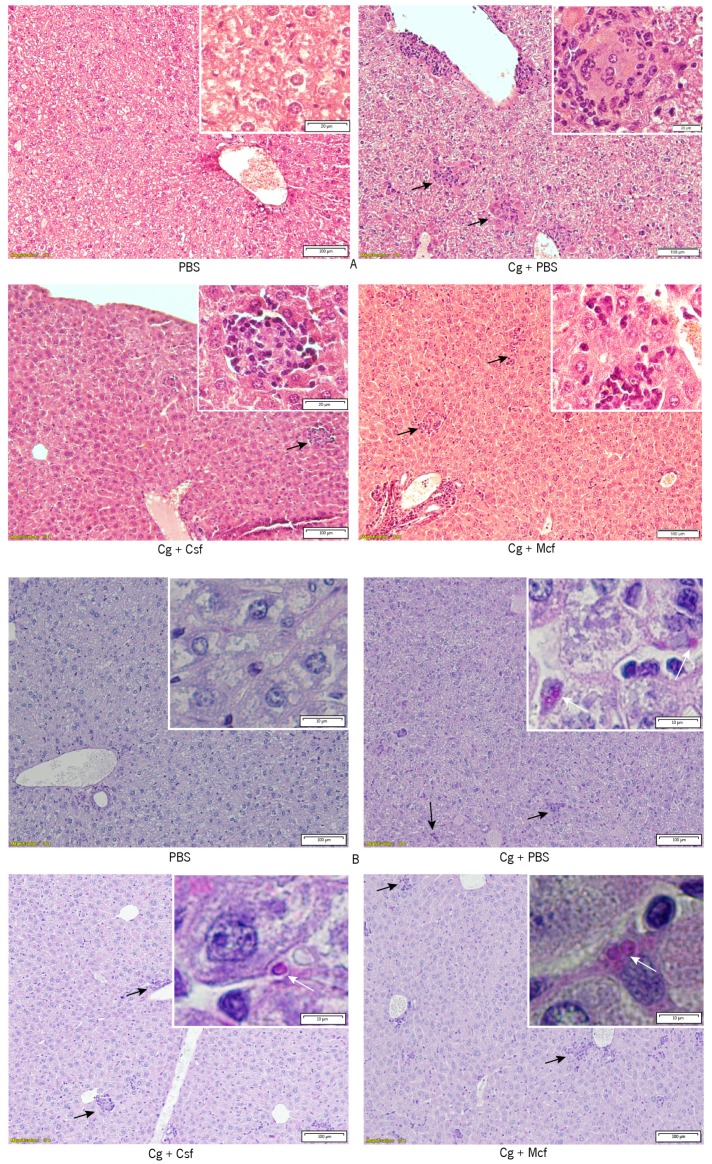
Analysis of liver histology in CD1 mice. (**A**) Representative hematoxylin-eosin and (**B**) periodic acid–Schiff (PAS)-stained examples of liver tissue from the indicated mouse groups. Black arrows denote inflammatory infiltrates that were mostly of the mononuclear type. Insets correspond to higher magnification micrographs. White arrows indicate PAS-stained *Candida glabrata* ATCC2001 cells. Scale bars are shown and apply to similar sized micrographs (100 μm) or insets (20 μm), as indicated.

Finally, liver and kidney histopathologies were analyzed in infected mice, as these organs are preferred targets in i.v. *Candida* spp. infections [45,86]. As could be expected, no yeasts were found in the non-challenged control groups, and their organs presented no significant histological alterations (Figure 6). Challenged mice showed inflammatory infiltrates in the liver, and less markedly in the kidneys (nontreated and treated groups, data not shown). The presence of polymorphonuclear cells was observed, but in general the infiltration remained mostly mononuclear. Yeasts were found in the liver (Figure 6), but not in the kidneys (data not shown) of treated and nontreated challenged groups. This fact corroborated the low CFU counts found in the kidneys.

## 4. Conclusions

In this work, a systemic infection exclusively originated from *C. glabrata* biofilm cells was simulated and a treatment evaluated. The observations here reported confirmed *C. glabrata* as a low inflammatory species and indicated that two-dose treatment with Csf and Mcf does not have a significant impact on liver and kidney fungal burden or recruited inflammatory infiltrate when mice are i.v. infected with *C. glabrata* biofilm-grown cells.
